# Compassion Satisfaction, Burnout, and Secondary Traumatic Stress among Saudi Nurses at Medical City: A Cross-Sectional Study

**DOI:** 10.3390/healthcare12080847

**Published:** 2024-04-17

**Authors:** Ahmad Rayani, Jean Hannan, Salman Alreshidi, Ahmad Aboshaiqah, Abdulaziz Alodhailah, Essa Hakamy

**Affiliations:** 1 Community and Psychiatric Mental Health Nursing Department, College of Nursing, King Saud University, Riyadh City 12372, Saudi Arabia; 2 Nicole Wertheim College of Nursing & Health Sciences, Florida International University, Miami, FL 33199, USA; jean.hannan@fiu.edu; 3 Department of Nursing Administration and Education, College of Nursing, King Saud University, Riyadh City 12372, Saudi Arabia; aaboshaiqah@ksu.edu.sa (A.A.);; 4 Department of Medical-Surgical Nursing, College of Nursing, King Saud University, Riyadh City 12372, Saudi Arabia

**Keywords:** compassion fatigue, Saudi nurses, compassion satisfaction, secondary traumatic stress

## Abstract

Compassion satisfaction, the pleasure gained from assisting others in their recovery from trauma, can help reduce the effects of burnout and secondary traumatic stress. As such, nurses’ job satisfaction can be increased by increasing compassion satisfaction and decreasing compassion fatigue. This study examined the incidence of compassion fatigue and other influencing variables, such as compassion satisfaction, burnout, and secondary traumatic stress, among Saudi nurses. This was a cross-sectional study using convenience sampling. Participants comprised 177 registered nurses from various nursing departments. Data collection included the Professional Quality of Life Scale based on lifestyle, demographic details, and occupation-related questions. The averages of scores for the variables, compassion satisfaction, burnout, and secondary traumatic stress, were recorded (37.1 ± 7.4, 25.7 ± 7.5, and 26.7 ± 6.4). Participants aged 36 or older comprised a negative factor for compassion satisfaction, while years of nursing experience and higher job satisfaction were favorable predictors. Together, these three variables accounted for 30.3% of the overall variation. Low job satisfaction and poor sleep negatively affected burnout, accounting for 39.8% of the total variance. The results offer insights into identifying the risks of compassion fatigue in nurses and help design strategies to address burnout and secondary traumatic stress while enhancing their compassion satisfaction levels.

## 1. Introduction

There has been an emergence of work-related stress among healthcare providers due to challenges such as time constraints, limited social support, rising patient expectations, and perceptions of low coping adequacy [1]. An association has often been found between reports of severe work-related stress among healthcare workers and the heavy emotional burden they bear from witnessing patients’ suffering [2]. A recent study revealed that a significant proportion of U.S. nurses (42%) rated their work environment as healthy. Nurses face severe safety and health-related concerns, such as acute and chronic effects of being overworked, as well as occupational stress [3]. Many researchers have found that nurses are particularly vulnerable to work-related stress [4,5]; in Saudi Arabia, for example, work-related stress affects 51.5% of nurses [6]. Work-related stress outcomes among nurses include reduced physical functioning, personal accomplishment, job satisfaction, emotional burnout, and high staff turnover [7,8,9]. There is an urgent need to thoroughly understand the factors that cause work-related stress in this group to design the necessary interventions and their subsequent implementation.

Compassion fatigue is the cost of caring and is defined as the stress response common among healthcare providers [7,10], which may considerably affect their ability to nurture [11]. The ability to nurture a significant source of work-related stress among healthcare providers was first evaluated among emergency nurses suffering burnout due to continuous exposure to distressing events and heavy workloads [11], where the variable compassion fatigue was included in the conceptual model and could be analyzed using the Professional Quality of Life Scale (ProQOL) [10,11,12]. The ProQOL comprises compassion satisfaction, burnout, and secondary traumatic stress subscales. Manifestations of burnout and secondary traumatic stress include poor physical and mental health statuses [13]. Recent estimates indicate that work-related stress affects 48.4–55% of healthcare providers [8,14], with billions lost in productivity annually in nations where this is experienced [15,16]. Moreover, between 36.8% and 78% of nurses are estimated to be at risk of compassion fatigue [17,18], which can affect their health, performance, job satisfaction, and staff turnover [19,20]. Consequently, compassion fatigue among nurses and other healthcare providers needs to be urgently addressed.

Burnout is a core component of compassion fatigue among nursing professionals. Symptoms vary widely, from depersonalization to feeling a lack of accomplishment, dissatisfaction with work, and emotional exhaustion [21]. The onset of these symptoms tends to be gradual as burnout occurs over time and generally lasts longer than compassion fatigue [22]. Burnout is seen among professionals of all types [23] but occurs in 38–64.0% of nurses [17,24]. It is crucial to diversify the study setting to identify and assess the condition among all work settings of this population. Furthermore, enhancing knowledge and understanding of the impact of occupational exposure-associated factors, such as experience, type of patient population, and department in which they work, can support strategy design to improve nurses’ overall well-being.

Secondary traumatic stress within the workplace, caused by secondary exposure to highly stressful or traumatic events [25], can also impact compassion fatigue, and a growing body of research has shown that this condition is common among nurses. An estimated 39.0–76.9% of nurses across various departments, particularly emergency, oncology, psychiatric, and pediatric departments, suffer from secondary traumatic stress [17,25,26]. Burnout severely negatively impacts healthcare providers’ ability to offer care and other profession-related tasks and interferes with exercising professional judgment [17]. Thus, researching secondary traumatic stress witnessed among the nursing population is critical.

Compassion fatigue can impair nurses’ physical and mental health. Compassion satisfaction—typically the sense of pleasure gained from supporting others in recovering from trauma [27]—can mitigate the effects of burnout and secondary traumatic stress [13]. Compassion satisfaction offers a sense of fulfillment and is based on nurses serving others to address their trauma and support their well-being, thereby doing their part in contributing to the organization [28]. Compassion satisfaction and job satisfaction differ slightly, where the former relates to emotional aspects, while the latter is the perception of the type of job, welfare at work, and other work-related skills [29]. Therefore, the study results convey the crucial role of compassion satisfaction in significantly reducing nurses’ turnover rates [27,30]. In contrast, stress exposure tends to increase compassion fatigue, a predictor of reduced compassion satisfaction [31]. Consequently, nurses’ job satisfaction can be improved by optimizing compassion satisfaction and reducing compassion fatigue.

A large body of research has examined the correlations that exist between burnout, compassion fatigue, and a variety of compassion satisfaction variables among different nursing and support professional populations in various geographical locations. Using a correlational design, one study on oncology nurses reported a negative correlation between compassion fatigue and compassion satisfaction, with a strong positive correlation found between burnout and compassion fatigue [32]. In New Zealand, surveys of palliative care nursing staff reported comparable results [33] as did a systematic review of mental health practitioners [34]. In the context of psychiatric nurses in Turkey, research identified a moderate correlation between compassion fatigue and compassion satisfaction with the positive relationship between these factors deemed insignificant [35]. Finally, compassion satisfaction and compassion fatigue were identified as the key determinants of burnout in a survey conducted on S.U.S. pediatric hematology oncology physicians [36].

Several stressors, such as long working hours, heavy workloads with a high expectation of high-quality care, and the requirement of healthcare reform initiatives, may influence Saudi nurses’ work lives. This situation is further escalated by conflicts and disputes between hospitals and patients [37,38]. Currently, research is at a nascent stage in Saudi Arabia, although the Arabic version of the ProQOL has been validated in some studies [39,40], and compassion fatigue among Saudi clinic nursing staff has attracted increasing attention. Given that compassion fatigue is frequently observed and has serious consequences, we hypothesize that female, old age, and critical care department will all be positive predictors of compassion fatigue burnout and negative predictors of compassion satisfaction. Moreover, greater nursing experience, better sleep quality, healthy lifestyle, and higher job satisfaction will be negative predictors of compassion fatigue burnout and positive predictors of compassion satisfaction.

This study aimed to investigate the status of this condition among Saudi clinic nurses. Specifically, this study seeks to answer the following questions:

1. What is the prevalence of compassion satisfaction, burnout, and secondary traumatic stress among nurses in Saudi Arabia?
2. What relationships are present among key variables such as compassion satisfaction, burnout, secondary traumatic stress, demographics, behavioral variables, and work-related share?

## 2. Materials and Methods

### 2.1. Study Sample and Design

This study employed a cross-sectional survey. Participants were recruited from the medical city in Riyadh, Saudi Arabia, from July to August 2023. The healthcare provider managed a medical city with 1200 licensed beds and approximately 2072 allied health staff who care for over 1,229,628 outpatients, admit 45,966 patients, and perform around 14,231 procedures every year [41]. The eligibility criteria included certified registered nurses with at least six months of clinical nursing experience, as transitioning from being newly employed to the first position can last at least this long. Participants were randomly selected from various departments by the nursing directors, including emergency, pediatrics, radiation, surgery, internal medicine, intensive care, and oncology. When considering the possibility of multiple regressions, a sample size of 137 was required based on the previously described effect size of 0.39 [42]. Small, medium, and large effect sizes are 0.15, 0.39, and 0.59, respectively. To achieve the minimum level of statistical power of 0.8, the model included 15 variables and a significance level of 0.05. 

### 2.2. Data Collection

The study was conducted in accordance with the Declaration of Helsinki, and prior to data collection, we obtained institutional review board approval from King Saud University Medical City (protocol code: KSU-HE-23-586; 6 June 2023). The selected data collection method involved a survey sent electronically via Google Forms to the directors of the nursing departments in the medical city. The electronic link to the survey explained the study’s aims and importance. The nursing director was asked to email approximately 200 surveys within his departments to the nursing staff emails in the randomly selected departments to achieve a minimum target sample of 150 nurses, with 177 responses and a response rate of 88.5%. A participant information sheet informed potential respondents that participation was voluntary, their responses would be anonymized, only interested individuals should volunteer to participate, and to protect their privacy, all related data will continue to be treated with strict confidentiality. After reading the participant information sheet, each participant signed an electronic consent form. 

The electronic data was kept in files stored on a password-protected computer or the secure college of nursing server behind the King Saud University. Only approved individuals directly related to the study had access to data. All the electronic data will be destroyed six years after the completion of the study.

### 2.3. Variables and Instruments

Two research instruments were used to gather data on work-related information, demographics, lifestyle factors, and dependent variables: compassion satisfaction, burnout, and secondary traumatic stress.

### 2.4. Questionnaire to Elicit Lifestyle, Work-Related, and Demographic Data

This survey collected participants’ demographic data such as age, gender, marital status, and educational background. Other work-related details included subdivision of employment, years of experience, number of working hours on average (calculated as the number of hours worked per week divided by the number of days worked per week), principal shifts worked, and job satisfaction ranking (ranging from 1 [very dissatisfied] to 5 [very satisfied]). Data regarding impactful lifestyle factors, such as length and quality of sleep (ranging from 1 [very poor] to 10 [perfect]), smoking status, and frequency of exercise per week, were also included. 

### 2.5. Professional Quality of Life Scale

The Arabic version of the ProQOL, Version 5, has been validated in the Arabic Language and uses a five-point Likert scale to assess the scores of the key variables, i.e., compassion satisfaction, burnout, and secondary traumatic stress, where 1 = never and 5 = very often [43]. The ProQOL comprises 30 items divided into subscales (compassion satisfaction, burnout, and secondary traumatic stress, with ten items each). The listed items 29, 17, 15, 4, and 1 were scored in reverse [12]. A higher score on the compassion satisfaction subscale indicated that the participants could provide adequate care. Conversely, a high burnout score indicated a higher likelihood of a burnout experience, and a high score on the secondary traumatic stress subscale indicated a need for respondents to examine their feelings about their job and work environment. For each subscale, a score of 22 or less, 23–41, and 42 and over indicated low, average, and high levels of compassion satisfaction, burnout, and secondary traumatic stress, respectively [12]. The construct reliability and validity of the ProQOL scale were verified by Stamm [12]. Stamm [12] employed both discriminant and convergent validity analysis techniques to convincingly show that each ProQOL subscale assesses a unique element. Importantly, it is not advisable to combine the scores of each subscale into a single score given that they function independently. The internal consistency of the three subscales of the ProQOL has been verified using psychometric evaluations, which reported Cronbach’s α reliability coefficients in the range of 0.84 to 0.90.

In this study, Cronbach’s coefficients for internal consistency reliability were 0.68 for the entire ProQOL 5, while the compassion satisfaction subscale scored 0.88, the burnout subscale 0.76, and the compassion fatigue subscale 0.85.

### 2.6. Statistical Analysis

The IBM Statistical Package for Social Sciences Version 28.0 was used. The prevalence of compassion satisfaction, burnout, and secondary trauma stress, along with the demographic and occupational features, used descriptive statistics. Variance homogeneity was measured by independent sample *t*-tests and ANOVA, in addition to Levene’s test, while the links between occupational and demographic variables were analyzed using Pearson’s or Spearman’s correlation analyses. This included the analysis of burnout, secondary trauma, and stress compassion satisfaction. The key variables were analyzed by the three multiple linear regression models from the demographics, lifestyle components, and work-related data collected. The level of significance was *p* < 0.05.

## 3. Results

### 3.1. Participant Characteristics

Table 1 illustrates the details of the participants, including their work-related characteristics, lifestyles, and demographics. Most participants were aged 26–35, female, married, and had a bachelor’s degree or higher in nursing. The predominant department among the participants was the medical department, with a significant number of individuals being general nurses. Regarding work experience, approximately 68 (38.9%) of the nurses had worked for six years or more. Regarding sleep patterns, 69 (39%) of the nurses reported sleeping more than six hours daily, while the majority 122 (68.9%) worked for more than eight hours daily, with 154 (87%) working during the day shift. Regarding smoking habits, only 41 (23.2%) of the nurses reported being smokers. On average, participants exercised for approximately 2.4 ± 2.1 days per week. The mean job satisfaction score was 2.3 ± 1.1, and the average sleep quality score was 1.9 ± 1.1.

### 3.2. Correlation between Compassion Satisfaction, Burnout, and Secondary Traumatic Stress

Table 2 displayed the Pearson correlations for compassion satisfaction, burnout, and secondary traumatic stress. Bivariate analysis revealed a strong negative association between burnout (*r* = −0.71, *p* < 0.001) and compassion satisfaction but a weak correlation with secondary traumatic stress (*r* = 0.27, *p* < 0.001). Burnout was positively associated with secondary traumatic stress (*r* = 0.56; *p* < 0.001). The compassion satisfaction, burnout, and secondary trauma stress scores included 37.1 ± 7.4, 26.7 ± 6.4, and 25.7 ± 7.5. Most participants, i.e., 117 (66.1%), reported average levels of compassion satisfaction, while at least 123 (69.5%) indicated moderate burnout.

### 3.3. Impact Factors of Compassion Satisfaction, Burnout, and Secondary Traumatic Stress 

The key variables were analyzed using independent sample *t*-tests and ANOVA. The sample of radiology nurses aged 36 years or older and those who smoked recorded less burnout (*p* < 0.05) (see Table 3). Quality of sleep had little effect on compassion satisfaction (*r* = 0.27, *p* < 0.001) but was weakly associated with burnout (*r* = −0.46, *p* <0.001) and secondary traumatic stress (*r* = −0.17, *p* = 0.020). However, job satisfaction had a stronger positive correlation with compassion satisfaction (*r* = 0.56, *p* < 0.001) and shared a moderately negative correlation with burnout (*r* = −0.59, *p* < 0.001) and secondary traumatic stress (*r* = −0.31, *p* < 0.001).

### 3.4. Regression Analysis and Covariates of Key Variables

The findings of regression analysis of the key variables, compassion satisfaction, burnout, and secondary traumatic stress, shared a statistically significant correlation and are listed in Table 4. The 30.3% variance in the compassion satisfaction model can be explained using the nurses’ age, experience, and job satisfaction. Specifically, age 36 or older negatively affected compassion satisfaction, while nursing experience and job satisfaction had a positive influence. Job satisfaction and sleep quality accounted for 39.8% of the total model variance and were both negatively associated with burnout. Job satisfaction was the only statistically significant factor affecting secondary traumatic stress, explaining 7.4% of the variance, and showed a negative relationship with secondary traumatic stress.

## 4. Discussion

Compassion satisfaction and fatigue are the two concepts of utmost importance for care delivery, department outcomes, and nurses’ health and safety. Caregiving brings about the gratification of compassion, and nurses experiencing high levels of compassion satisfaction tend to feel content and cheerful, enabling them to devote more energy to their work. Conversely, compassion fatigue frequently includes burnout and secondary traumatic stress and can cause nurses to consider leaving their employment.

In this study, 177 Saudi nurses were assessed for burnout, compassion satisfaction, and secondary traumatic stress using the ProQOL Version 5 questionnaire. The average scores for compassion satisfaction, burnout, and secondary stress included 37.1 ± 7.4, 26.7 ± 6.4, and 25.7 ± 7.5. Compared to previous studies, our study revealed similar scores for compassion satisfaction and relatively higher burnout and secondary stress [44,45]. Also, the study sample of Saudi nurses exhibited higher secondary stress and burnout than those in four public hospitals in Portugal and emergency department nurses in the U.S. [4,46]. Disparities in work environments, workload, unit culture, and nursing traits may account for the observed differences. Based on the available evidence, Saudi clinical nurses may suffer from compassion fatigue. Therefore, health organizations should prioritize measures to assist nurses in coping with burnout and secondary traumatic stress. Furthermore, an increase in the number of years of nursing experience and job satisfaction were significant predictors of compassion satisfaction. Moreover, age 36 or higher was a negative predictor of compassion satisfaction. In addition, job satisfaction was a negative predicator of compassion fatigue and burnout, whereas good quality of sleep was a negative predictor of burnout. However, female gender, different departments, and healthy lifestyle were not found to be predictors of any of the three criterion variables, contrary to our hypotheses.

Our findings suggest that greater nursing experience and job satisfaction positively impact compassion satisfaction levels. Previous studies have also indicated that nurses with higher clinical experience levels, social support, life satisfaction, and job satisfaction are highly likely to record more compassion satisfaction [47,48]. The development of compassion satisfaction can be influenced by subjective feelings of competence and fulfillment regarding one’s career [49]. Additionally, Teffo et al. [50] identified years of employment and factors such as finding work exciting, believing in one’s ability to make a difference, enjoying interactions with coworkers, and work experience as predictors of compassion fulfillment.

Future research would focus on factors that contribute to job satisfaction, such as increased role autonomy and manageable workloads, as well as interventions to improve sleep quality, such as sleep hygiene therapies. By addressing these aspects, healthcare organizations can create a more supportive environment for nurses, thereby furthering the effect of compassion satisfaction and reducing compassion fatigue.

Job satisfaction and sleep quality were identified as statistically significant determinants of burnout, as evidenced by the multiple linear regression models. These findings align with a similar study conducted among emergency room medical staff in Western Turkey, which revealed a strong inverse relationship between burnout and job satisfaction. Moreover, this study found that “personal accomplishment” and “emotional exhaustion” were two aspects of burnout that had a considerable negative impact on job satisfaction [29]. The current findings are similar to those of a study on Korean nurses who specialized in handling tuberculosis patients [51] but differ from those on Iranian primary healthcare personnel [52]. These variations in findings may be attributed to different sociocultural factors that influence nurses’ experiences in different regions.

Higher job satisfaction and maintaining a healthy lifestyle appear to be crucial characteristics contributing to Saudi nurses feeling secure, supported, and protected from negative thoughts about their workplace. Toppinen-Tanner et al. [53] revealed their study results that occupation-related training to manage stress and cognitive behavioral therapy along with social support could significantly positively affect their overall satisfaction by reducing nurses’ burnout. 

Considering the caregiver role and many interactions between nurses and ailing patients, they are more vulnerable to secondary traumatic stress. This study found a correlation between secondary traumatic stress and job satisfaction among nurses. Previous research has also indicated that nurses with secondary traumatic stress often experience sleep difficulties and work longer hours [54].

Secondary traumatic stress is positively affected by job satisfaction and is considered an indicator [55]. Nurses with secondary traumatic stress may be more inclined to contemplate changing careers or resorting to alcohol to cope with job-related stress [56]. Hence, the prevalence of secondary traumatic stress among nurses at a greater risk should be assessed as early as possible to adequately support them and design targeted interventions. Previous studies have highlighted the influence of the department on the stress levels of nurses, particularly those in oncology, I.C.U., psychiatric, emergency, and pediatric departments, who are highly likely to experience burnout, fatigue, and secondary traumatic stress [8,44,48]. Moreover, among Swiss physicians, it was reported that working in rural areas can be a risk factor for burnout [57]. In contrast, employment in rural areas was not found to predict burnout among general nurses and practitioners in Spain [58].

However, the current findings do not convey statistically significant differences in these factors across different departments. This aligns with some previous research but may be influenced by differences in the sampled populations from tertiary hospitals in the central region of Saudi Arabia [45,59]. Nevertheless, the current study emphasizes the low professional quality of life of Saudi nurses.

This study has some limitations. First, the cross-sectional study design limits the follow-up of changes in nurses’ work–life quality. A longitudinal approach would have provided valuable insights into how the variables of interest evolved over a longer period. Furthermore, the study had a limited sample size, involving 2072 nurses from medical city; however, only 177 nurses responded to the survey. This low response rate (approximately 8.5%) may have introduced a response bias and reduced the overall representativeness of the findings. The sampling method used here is that of convenience, restricting generalizability to other populations. The findings can only be generalized to nurses working in the medical city in Saudi Arabia and may not apply to a broader population of nurses. Finally, relying on self-reported instruments for data collection could potentially influence reliability. To enhance the generalizability of the results, future studies should employ a combination of sampling methods, including random and stratified sampling, to ensure a more diverse and representative sample of nurses from various hospitals and regions.

### Implications for Practice

The ever-increasing need for health practitioners demands a commensurate increase in research into the ongoing pressures placed on nursing professionals. Numerous strategies have been proposed for use in clinical settings. A key implication of the current work for professional healthcare practice is the potentially positive effect of holding open discussions with administration and management in the workplace on the subject of compassion fatigue. Being able to freely discuss the emotional toll of clinical care can be cathartic for practitioners. Additionally, not having such discussions can have a markedly detrimental effect on healthcare workers. To illustrate, professionals working in trauma settings with high-risk populations can suffer secondary trauma when they are unable to appropriately process their exposure to traumatic situations. Correspondingly, it is also vital that employers encourage the uptake of continued self-care activities by their staff. This process does not need to be complicated and could simply involve encouraging staff to take regular breaks throughout their workday and eat lunch away from their desks. Healthcare professionals often feel overwhelmed by the many tasks they need to complete in a day, from compiling progress notes, attending meetings, responding to emails, and managing crisis phone calls to completing required training, tracking productivity, and consulting with a variety of colleagues and departments.

The extremely high workload of those in the healthcare profession often leads many healthcare practitioners to prioritize work tasks at the expense of their needs. This includes sacrificing breaks and lunch hours to meet deadlines and hit productivity targets. A significant reduction in workplace burnout and compassion fatigue could be achieved if, instead of pressuring staff to complete work tasks, supervisors and managers encouraged staff to engage in self-care and balance their work obligations with their needs.

Importantly, the practice of self-care is not confined to the workplace. Healthcare employers should also encourage their staff to engage in self-care activities outside working hours. The adoption of effective coping strategies by healthcare workers can help them to avoid compassion fatigue and burnout. Some strategies include participating in leisure activities such as team sports, spiritual practices such as meditation or prayer, relaxation activities such as napping in the afternoon, listening to or playing music, or watching a favorite television program, and increasing physical exercise, for example, by taking up running, cycling, or other physical activities. These strategies can help healthcare professionals to safeguard themselves against the effects of secondary trauma and compassion fatigue.

## 5. Conclusions

Our research demonstrated that nurses integrated within the medical city showed comparable levels of compassion satisfaction but higher levels of secondary traumatic stress and burnout than those in the past. These scores were strongly associated with various demographic, occupational, and behavioral variables. Notably, three correlations stood out as particularly significant. 

Compassion satisfaction, burnout, and secondary traumatic stress were negatively correlated. This suggests that compassion satisfaction negates the influence of burnout and secondary traumatic stress. Second, our research revealed a negative correlation between burnout levels and sleep quality among nurses. This indicates that higher burnout levels are associated with poorer sleep quality, highlighting the importance of addressing burnout to promote better sleep and overall well-being. Third, we observed a positive association between compassion satisfaction and nursing experience. Nurses with more experience expressed higher levels of compassion satisfaction, indicating that tenure positively affected this variable. 

Thus, the aim should be to design targeted interventions and address coping strategies for all high-risk populations, nurses in this study, to mitigate burnout and stress. Addressing these issues could foster compassion satisfaction and improve nurses’ overall well-being. Innovative programs tailored to Saudi nursing settings should be considered to support this essential workforce in providing high-quality patient care. These findings may assist the development of specialized mediations and support mechanisms to promote nurses’ psychological well-being in the medical city healthcare setting.

The findings of the present research suggest a number of productive avenues for further research to develop our understanding of nursing. For example, a mixed method research approach could be adopted to gather more robust findings. This approach is advisable as it provides researchers with the flexibility to utilize various study designs, such as observational and randomized trial studies. Moreover, a more comprehensive study could be achieved through the use of surveys designed to capture data on the impact of compassion fatigue, burnout, and compassion satisfaction, as well as recommendations for improvements directly from nursing staff. Furthermore, it is important to note that this study is concerned with examining compassion fatigue, burnout, and compassion satisfaction in the context of urban hospitals. Future studies could explore these elements in other settings, such as hospices, private practices, universities, schools, and child protective environments. This would enhance and broaden our understanding of compassion fatigue, burnout, and compassion satisfaction in the nursing profession and their implications for healthcare professionals. 

## Figures and Tables

**Table 1:** Demographic characteristics of the participants (*N* = 177).

Variable	Category	n	%
Age (years)			
	18–25	36	20.3
	26–35	112	63.3
	36–56	29	16.4
Gender			
	Male	82	46.3
	Female	95	53.7
Marital status			
	Married	98	55.4
	Unmarried	79	44.6
Level of education			
	Associate degree or less	17	9.6
	Bachelor’s degree or above	160	90.4
Department			
	Emergency department	27	15.3
	Pediatrics department	14	7.9
	Radiation department	5	2.8
	Surgical department	35	19.8
	Medical department	57	32.1
	Oncology department	12	6.8
	Intensive care unit	27	15.3
Years of nursing experience			
	≤1 year	34	19.2
	2 to 5 years	73	41.2
	6 to 10 years	39	22.3
	>11 years	29	16.6
Working position			
	General nurse	151	85.3
	Head nurse	16	14.7
Average work hours per day			
	≤8 h	55	31.1
	>8 h	122	68.9
Predominantly worked shift			
	Days	154	87
	Nights	23	13
Smoking			
	Yes	41	23.2
	No	136	76.8
Sleep hours per day			
	≤6 h	108	61
	>6 h	69	39

**Table 2:** Correlations between compassion satisfaction, burnout, and secondary traumatic stress.

Variables	Compassion Satisfaction	Burnout	Secondary Traumatic Stress
Compassion satisfaction	-		
Burnout	−0.71 *	-	
Secondary traumatic stress	−0.27 *	0.56 *	-
Mean	37.17	26.76	25.74
Standard deviation	7.41	6.43	7.55

* *p* < 0.001.

**Table 3:** Univariate analyses of the factors associated with compassion satisfaction, compassion fatigue, and burnout (*N* = 177).

Variable	Category	Compassion Satisfaction			Burnout			Secondary Traumatic Stress		
		Mean (SD)	t/F	*p*	Mean (SD)	t/F	*p*	Mean (SD)	t/F	*p*
Age (years)	18–25	36.41 (8.71)	0.28	0.750	29.33 (6.59)	4.230	0.016	26.02 (7.31)	1.116	0.330
	26–35	37.47 (6.99)			26.37 (6.17)			26.14 (7.61)		
	>36	36.96 (7.45)			25.06 (6.54)			23.82 (7.56)		
Gender	Male	37.56 (7.10)	0.412	0.522	26.50 (5.70)	0.254	0.615	25.79 (7.40)	0.007	0.932
	Female	36.84 (7.70)			26.98 (7.02)			25.69 (7.71)		
Marital status	Married	37.40 (7.61)	0.136	0.712	26.22 (6.60)	0.986	0.322	25.96 (7.26)	0.123	0.727
	Unmarried	36.98 (7.29)			27.19 (6.29)			25.56 (7.80)		
Level of education	Associate degree or less	39.29 (7.20)	1.539	0.216	24.58 (7.78)	2.162	0.143	23.41 (5.20)	1.795	0.182
	Bachelor’s degree or above	36.95 (7.42)			26.99 (6.25)			25.98 (7.73)		
Department	Emergency department	36.81 (7.63)	1.536	0.169	28.11 (6.26)	2.421	0.029	28.48 (7.90)	1.760	0.110
	Pediatrics department	34.81 (5.60)			30.07 (5.71)			27.14 (6.37)		
	Radiation department	40.20 (4.60)			21.00 (3.08)			21.20 (6.64)		
	Surgical department	38.22 (7.25)			25.54 (6.76)			25.68 (7.78)		
	Medical department	36.01 (7.94)			27.29 (6.45)			25.68 (7.12)		
	Oncology department	35.41 (6.69)			28.00 (5.22)			26.91 (7.98)		
	Intensive care unit	40.03 (7.24)			24.66 (6.34)			22.77 (7.67)		
Years of nursing experience	≤1 year	36.70 (8.31)	0.886	0.460	28.11 (6.03)	1.985	0.118	24.79 (7.83)	1.364	0.256
	2 to 5 years	36.46 (7.18)			27.41 (6.46)			27.01 (7.87)		
	6 to 10 years	37.48 (7.59)			25.66 (6.48)			25.20 (6.29)		
	>11 years	39.00 (6.88)			24.86 (6.57)			24.13 (7.99)		
Working position	General nurse	37.09 (7.57)	0.126	0.723	26.96 (6.38)	1.035	0.310	25.81 (7.46)	0.099	0.753
	Head nurse	37.65 (6.51)			25.57 (6.70)			25.30 (8.18)		
Average work hours per day	≤8 h	37.38 (6.26)	0.271	0.393	26.07 (5.56)	0.917	0.340	26.43 (8.00)	0.677	0.412
	>8 h	37.08 (7.90)			27.07 (6.78)			25.42 (7.35)		
Predominantly worked shift	Days	37.18 (7.54)	0.004	0.951	26.54 (6.33)	1.354	0.246	25.39 (7.44)	2.479	0.117
	Nights	37.08 (6.66)			28.21 (7.07)			28.04 (8.06		
Smoking	Yes	38.09 (6.20)	0.824	0.365	24.63 (5.72)	6.004	0.015	23.92 (7.15)	3.112	0.079
	No	36.89 (7.74)			27.40 (6.51)			26.28 (7.60)		
Second-hand smoking	Yes	37.80 (6.78)	1.162	0.283	25.57 (7.39)	0.076	0.783	25.57 (7.39)	0.076	0.783
	No	36.59 (7.94)			25.89 (7.73)			25.89 (7.73)		
Sleep hours per day	≤6 h	36.94 (7.24)	0.267	0.606	26.99 (6.37)	0.347	0.557	25.95 (7.91)	0.221	0.639
	>6 h	37.53 (7.41)			26.40 (6.55)			25.40 (6.99)		

**Table 4:** Regression analysis examining covariates of CS, BO, and STS (*N* = 177).

	Compassion Satisfaction ^a^					Burnout ^b^					Secondary Traumatic Stress ^c^				
Model	B	SE	Beta	t	*p*	B	SE	Beta	t	*p*	B	SE	Beta	t	*p*
(Constant)	26.365	2.879	-	9.158	0.000	38.127	2.321	-	16.427	0.000	30.980	3.380	-	9.166	0.000
Age = 36 or higher	−2.993	1.230	−0.243	−2.434	0.016	-	-	-	-	-	-	-	-	-	-
Years of nursing experience	2.576	0.923	0.338	2.790	0.006	-	-	-	-	-	-	-	-	-	-
Job satisfaction	4.120	0.523	0.584	7.874	0.000	−3.034	0.422	−0.496	−7.193	0.000	−2.193	0.614	−0.305	−3.571	0.000
Sleep quality	-	-	-	-	-	−1.525	0.418	−0.255	−3.651	0.000	-	-	-	-	-

^a^: F = 5.442, *p* = 0.000, R^2^ = 0.371, Adjusted R^2^ = 0.303. ^b^: F = 7.768, *p* = 0.000, R^2^ = 0.457, Adjusted R^2^ = 0.398. ^c^: F = 1.818, *p* = 0.030, R^2^ = 0.164, Adjusted R^2^ = 0.074.

## Data Availability

Data will be made available upon request to the corresponding author.
